# Urine high–sensitive troponin I in children cannot offer an applicable alternative to serum

**DOI:** 10.3389/fcvm.2024.1391434

**Published:** 2024-05-20

**Authors:** Matija Bakoš, Daniel Dilber, Anamarija Jazbec, Tomo Svaguša, Ana-Meyra Potkonjak, Duje Braovac, Željko Đurić, Andrea Radeljak, Ana Lončar Vrančić, Hrvoje Vraneš, Slobodan Galić, Milivoj Novak, Ingrid Prkacin

**Affiliations:** ^1^ Department of Pediatrics, University Hospital Centre Zagreb, Zagreb, Croatia; ^2^ School of Medicine, University of Zagreb, Zagreb, Croatia; ^3^ Department for Forest Inventory, Management Planning and Remote Sensing, University of Zagreb Faculty of Forestry and Wood Technology, Zagreb, Croatia; ^4^ Department of Cardiology, Dubrava University Hospital, Zagreb, Croatia; ^5^ Department of Gynecology and Obstetrics, Sestre Milosrdnice University Hospital Centre, Zagreb, Croatia; ^6^ Department of Cardiac Surgery, University Hospital Centre Zagreb, Zagreb, Croatia; ^7^ Department of Medical Biochemistry and Laboratory Medicine, Merkur University Hospital, Zagreb, Croatia; ^8^ Department of Laboratory Diagnostics, University Hospital Centre Zagreb, Zagreb, Croatia; ^9^ Department of Internal Medicine, Merkur Clinical Hospital Zagreb, Zagreb, Croatia

**Keywords:** high-sensitive troponin I, cardiac troponin I, urine, serum, cardiac surgery

## Abstract

**Introduction:**

In children, congenital heart defects represent the primary cause of increased serum troponin I. The elimination process of cardiac troponin I from the bloodstream and the factors influencing this process remain unknown. The objective of this study was to explore the role of troponin I as an indicator of cardiac damage in children both in serum and urine, a concept previously investigated in adults.

**Methods:**

Our prospective study involved 70 children under 24 months of age. The first group underwent ventricular septal defect repair, while the second group involved children who had undergone partial cavopulmonary anastomosis. For these groups, urine and serum troponin I were assessed on four occasions. The third group, consisting of healthy children, underwent a single measurement of urine troponin I.

**Results:**

Serum troponin I values exhibited an expected elevation in the early postoperative period, followed by a return to lower levels. Significantly higher concentrations of serum troponin I were observed in the first group of children (*p* < 0.05). A positive correlation was found between troponin I in the first three measurements and cardiopulmonary bypass and aortic cross-clamping time. There was no discernible increase in urine troponin I directly related to myocardial damage; troponin I couldn't be detected in most urine samples.

**Discussion:**

The inability to detect troponin I in urine remains unexplained. Potential explanatory factors may include the isoelectric point of troponin I, elevated urinary concentrations of salts and urea, variations in urine acidity (different pH levels), and a relatively low protein concentration in urine.

## 1 Introduction

In children, congenital heart defects (CHD) represent the primary cause of increased serum troponin I (cTnI). The distinct hemodynamic conditions of CHD include volume and pressure overload, cyanosis, and pulmonary hypertension, each of which can lead to necrosis, apoptosis, and mechanical stress, ultimately resulting in cardiac damage (1). High-sensitive troponin I (hsTnI), as one of the markers used to detect cardiac damage in children, exists in two isoforms: adult troponin I and an isoform similar to adult skeletal troponin I. The latter is completely replaced by the adult isoform by the age of nine months in infants without CHD. In children with CHD, this replacement occurs somewhat later, usually by the second year of life (2, 3). Although postoperative serum troponin I values and their trends after cardiac surgery in children are well-documented, there is a limited number of studies incorporating the latest type of hsTnI test (4–6). Research on serum troponin I has been conducted, but there are no published studies examining the renal elimination of troponin I in children, unlike in adults (7–10). The elimination process of cardiac troponin I (cTnI) from the bloodstream and the factors influencing this process remain unknown (11). Three proposed elimination mechanisms of cTnI from the bloodstream include: (a) involvement of the reticuloendothelial system and intracellular cleavage (11, 12), (b) the direct effect of proteolytic enzymes in the blood (11, 13, 14), and (c) the elimination of cTnI molecules through urine or oral fluid (11, 15, 16). These mechanisms are interconnected, influencing one another. After being released from the heart, proteolytic enzymes break down the cTnI molecule into smaller fragments which can be removed through glomerular filtration (8, 9, 16). Recent renal detection of cTnI has shown promising results for cardiovascular assessment in adults (7). In one such study, the authors demonstrated that urine hsTnI could be a viable biomarker for predicting subsequent cardiovascular events in patients with diabetes mellitus (7). Chronic renal failure can lead to elevated serum hsTnI values without myocardial damage (16). Understanding the elimination mechanism is crucial for avoiding false positive or negative findings.

The objective of this study was to examine the concentration of hsTnI in both serum and urine after cardiac surgery, comparing them to urine samples of healthy children. The aim was to contribute to the complexity of understanding the elimination process of cTnI. Consequently, a noninvasive urine troponin I test could be developed to detect myocardial damage.

## 2 Materials and methods

### 2.1 Population

This study was conducted at the University Hospital Centre (UHC) Zagreb, Zagreb, Croatia, involving 70 children under the 24 months of age (55 male, 25 female) from November 2014 to August 2021. Various patient data were gathered encompassing demographic, clinical, and details regarding the duration of hospital and pediatric intensive care unit (PICU) stays, mechanical ventilation duration, mortality, morbidity, and surgical-related information such as the time of cardiopulmonary bypass and time of aortic cross-clamp.

Inclusion criteria comprised children aged up to 24 months who underwent surgical closure of ventricular septal defect (VSD) or surgical formation of bidirectional cavopulmonary anastomosis (BCPC). Healthy children included in the study were those without CHD and other acute or chronic diseases.

Exclusion criteria involved the presence of a syndrome of known etiology and incomplete data, which led to exclusion from the study. Healthy subjects were excluded if the examination revealed other known acute and/or chronic diseases.

Written informed parental consent was obtained for all participating patients.

The study included three groups of children: the first group had undergone surgical VSD closure, the second group had undergone BCPC surgery, and the third group consisted of healthy children. In the first two groups, cTnI levels were measured at four time points: the day before cardiac surgery (measurement 1), immediately after surgery (measurement 2), on the first postoperative day (measurement 3), and on the third postoperative day (measurement 4). In the third group, the first morning urine was analyzed only once. For ethical reasons, blood samples were not taken and analyzed from healthy children.

Due to the dependence of urinary biomarker concentration on both the urinary flow rate and the excretion rate of the biomarker, it is common to express urinary biomarker concentrations as a ratio to the urinary creatinine concentration. The creatinine concentration was also analyzed in all the previously mentioned samples.

### 2.2 Sample collection

Immediately after withdrawing blood samples from each individual subject, serum was separated from the blood, and was stored at the temperature of −20°C. After the samples of all subjects were collected, the concentration of hsTnI were determined on the analytical system immunochemistry analyzer Abbott Architect i1000SR (Abbott Laboratories) using chemiluminescent immunoassay on microparticles (CMIA, ARCHITECT STAT high-sensitive Troponin-I), accredited according to HR EN ISO 15189:2012. According to the manufacturer, this assay uses a sample volume of 160 µl.

### 2.3 Troponin I analysis

Two steps of analysis are present. First, cTnI from the sample binds to the specific mouse monoclonal anti-cTnI (capture) antibody-coated microparticles. The epitope binding specificity is directed against amino acids 24–40 on the cTnI protein. A second, specific mouse-human chimeric, monoclonal anti-troponin I acridinium-labeled antibody acts as a detection antibody. This chimeric antibody, which has an epitope binding specificity directed against amino acids 41–49 on the cTnI molecule, was designed to reduce susceptibility to heterophilic antibody interference (17). Abbott uses calibrators prepared gravimetrically using recombinant human cardiac troponin IC complex to determine hsTnI. Results are reported in ng/L. According to the manufacturer, applicable samples for determination of hsTnI are serum or plasma. The method has not been validated for other sample types. Therefore, before analyzing the urine samples, we had to perform a verification of the method specifications declared by the manufacturer to make sure that the method is acceptable for the determination of hsTnI concentration in urine. In the verification process, we used a urine sample with a very low concentration of hsTnI (<1 ng/L), which was spiked with a commercially available sample with declared concentration of hsTnI. Thus, we obtained samples with suitable matrix for verification. The verification of precision was carried out in accordance with the manufacturer's declaration. The verification of precision was carried out in accordance with the CLSI EP15-A guidelines. The obtained results were in accordance with the manufacturer's declaration. Evaluation of detection capability was performed according CLSI EP17-A2 guidelines. Limits of blank, detection, and quantitation were confirmed in accordance with the guideline's requirements (30 measurements over three days, with a minimum of 87% of the samples meeting the set limit). We confirmed the limit of blank <1 ng/L, the limit of detection at 2 ng/L and the limit of quantification at 5 ng/L.

### 2.4 Statistical analysis

Descriptive statistics were done for all variables analyzed in the study. A significance level of 5% (*p* < 0.05) was considered statistically significant, unless otherwise stated for all statistical tests,

Differences between demographic data between all three groups were tested by analysis of variance (ANOVA) and Tukey's *post hoc* test was used if there was a statistically significant difference between the groups. Further statistics were performed on the first two investigated groups, due to ethical concerns regarding a blood sampling from healthy children. Differences in PICU stay, hospital stay and cardiopulmonary bypass time (CPB) between Groups 1 and 2 were tested using rank Wilcoxon Mann–Whitney test. The troponin I difference in serum between groups (1 and 2) for each individual measurement was tested using a one-sided Wilcoxon Mann–Whitney test, considering higher values of hsTnI were observed in group 1. RM ANOVA was used to test the difference in troponin I in serum between the Groups during the analyzed period (measurements, days) and to explore the interaction between groups and days, Given that one of the assumptions for RM ANOVA is the normal distribution of troponin I in serum, troponin I levels were logarithmically transformed (ln transformation) due to the non-normality of the distribution. In cases where the assumption of sphericity was not met, adjustments with Greenhouse-Geisser Epsilon were applied. For both combined groups, the correlation between creatinine clearance, PICU stay, hospital stay, mechanical ventilation, CPB, aortic-cross clamping (AOX), and lnTroponinI levels in serum was investigated for each analyzed day.

All statistical analyzes were performed in the statistical package SAS9.4.

## 3 Results

### 3.1 Demographic and clinical data

Patients were categorized into three groups. The first group comprised 38 children with VSD who underwent cardiac surgery. The second group included 9 infants before and after the surgical formation of BCPC. The third group consisted of a total of 23 healthy children. Demographic and clinical data are shown in Table 1. In terms of age, the first group exhibits a statistical difference in age compared to the second group, whereas regarding weight and body surface area (BSA), the difference is observed between the second and third groups (Table 1). The inability to obtain serum troponin samples from the population of healthy children hindered a thorough examination of all remaining clinical indicators. Consequently, additional analyses were conducted on the initial two examined groups (Tables 2–5). PICU stay (*p* < 0.001), and hospital stay (*p* = 0.054) were significantly higher in the second group compared to the first, while there was no difference in CPB time between the first and second group (*p* = 0.525), which is shown in Table 2.

**Table 1:** Demographic and clinical data of patients included in the study.

Variables	Groups	*N*	Mean	SD	Median	IQR (1–3)	ANOVA
Age (months)	1	38	9.1	4.8	7	6–13	F = 3.98
	2	9	4.8	1.1	4.5	4–6	***p* = 0.023**
	3	23	7.5	3.9	7	5.5–9	(1,3)(2,3)
Weight (kg)	1	38	7.1	2.1	7	5.4–8	F = 5.07
	2	9	5.9	0.9	6.1	5.3–6.3	***p* = 0.009**
	3	23	8.2	1.7	8.2	7.1–9	(1,2)(1,3)
Height (cm)	1	38	69.4	8.1	69	64–74	F = 1.83
	2	9	64.4	3.6	64	62.5–68	*p* = 0.168
	3	23	70.1	8.5	70	66–72	
BSA (m^2^)	1	38	0.36	0.07	0.37	0.31–0.40	F = 3.99
	2	9	0.33	0.03	0.33	0.30–0.34	***p* = 0.023**
	3	23	0.39	0.06	0.41	0.36–0.42	(1,2)(1,3)

*N*, number; SD, standard deviation; IQR, interquartile range; ANOVA, analysis of variance; Group 1, patients after VSD surgery; Group 2, patients after formation of BCPC anastomosis; Group 3, healthy control; kg, kilograms; cm, centimeters; m^2^, square meters.

Bold values represent statistically significant “*p*” value.

**Table 2:** Comparison of clinical data between two groups.

Variables	Groups	*N*	Mean	SD	Median	IQR (1–3)	WMW
PICU stay (d)	1	38	5.4	4.6	4	3–5	*S* = 39.8
	2	9	11.2	5.5	10	7–14	***p* = 0.0008**
Hospital stay (d)	1	38	21.6	13.7	16	13–26	*S* = 287.5
	2	9	37.8	37.6	22	18–37	*p* = 0.054
Mechanical ventilation (min)	1	38	39.9	64.3	9	6–44	*S* = 248
	2	9	93	126.2	68	4–407	*p* = 0.393
CPB time (min)	1	38	108.7	30.9	103	89–127	*S* = 240
	2	9	112.2	35.6	123	80–139	*p* = 0.525
AOX time (min)	1	38	63.5	21.9	59	50–74	

*N*, number; SD, standard deviation; IQR, interquartile range; WMW, Wilcoxon Mann–Whitney test; Group 1, patients after VSD surgery; Group 2, patients after formation of BCPC anastomosis; d, days; min, minutes; CPB, cardiopulmonary bypass time; AOX, aortic cross-clamping time.

Bold values represent statistically significant “*p*” value.

**Table 3:** Results of one-sided Wilcoxon Mann–Whitney two sample test for serum troponin I for each measurement.

	Troponin I in serum (ng/L) (median and IQR)			
Measurement	1	2	3	4
Group 1	11.8 (3.9–21.6)	13,322.5 (7,118–23,737.5)	5,735 (3,163–8,216)	1,164 (526.3–2,529)
Group 2	10.6 (6.4–18)	6,905.7 (3,083–11,780)	2,267.4 (1,226–8,298)	868 (649–4,167)
WMW test	*S* = 141.50	*S* = 139.00	*S* = 174.00	*S* = 160.00
One sided Pr > *S*	*p* = 0.482	***p* = 0.040**	*p* = 0.181	*p* = 0.429

IQR, interquartile range; Group 1, patients after VSD surgery; Group 2, patients after formation of BCPC anastomosis; S, S statistics; WMW, Wilcoxon Mann–Whitney test.

Bold values represent statistically significant “*p*” value.

**Table 4:** Results of RM ANOVA for serum lnTroponinI during the analyzed period.

Source of variability	df.	Mean square	*F*-value	*p* > *F*
Group	1	0.0577637	0.02	0.9031
Error	30	3.8333819		
Time	3	188.6416853	92.01	**<0** **.** **0001** **^a^**
Time × group	3	0.1549593	0.08	0.9519
Error (time)	90	2.0501904		
Sphericity tests	5		Chi^2^ = 37.02	**<0** **.** **0001**

lnTroponinI, logarithmic transformation of troponin I; df., degree of freedom; Chi^2^, chi-squared test.

^a^ Adjustment with Greenhouse-Geisser Epsilon = 0.8036.

Bold values represent statistically significant “*p*” value.

**Table 5:** Correlations between logarithmic transformation of serum troponin I (lnTroponinI) values and analyzed variables for both groups.

		Logarithmic transformation of serum troponin I values			
		Measurements			
Variables	*	1	2	3	4
CrCl by meas.	*r*	−0.259	−0.003	−0.108	0.121
	*p* > |*r*|	0.111	0.982	0.470	0.422
PICU stay	*r*	0.105	0.115	0.221	−0.253
	*p* > |*r*|	0.524	0.463	0.144	0.102
Hospital stay	*r*	0.177	0.041	0.023	−0.121
	*p* > |*r*|	0.280	0.792	0.883	0.442
Mechanical ventilation	*r*	0.262	0.076	0.159	−0.126
	*p* > |*r*|	0.106	0.629	0.296	0.420
CPB	*r*	0.074	0.378	0.397	−0.161
	*p* > |*r*|	0.654	0.013	0.007	0.303
AOX (group 1)	*r*	0.224	0.302	0.374	−0.108
	*p* > |*r*|	0.217	0.083	0.025	0.532

* *r*, pearson correlation coefficient; *p*, probability under Ho:*ρ* = 0, CrCl, creatinine clearance; meas., measurement; PICU, pediatric intensive care unit; CPB, cardiopulmonary bypass time; AOX, aortic cross – clamping time.

### 3.2 Serum cTnI

In the first group, the median value of cTnI in serum before surgery was 11.8 [IQR: 3.9–21.6] ng/L (Table 3). Immediately after the surgery (measurement 2) the median value of cTnI in serum was 13 322.5 [IQR: 7,118–23 737.5] ng/L. On the first postoperative day the median value was 5,735 [IQR: 3,163–8,216] ng/L, and on the third postoperative day the median value was 1,164 [IQR: 526.3–2,529] ng/L.

**Figure 1:**
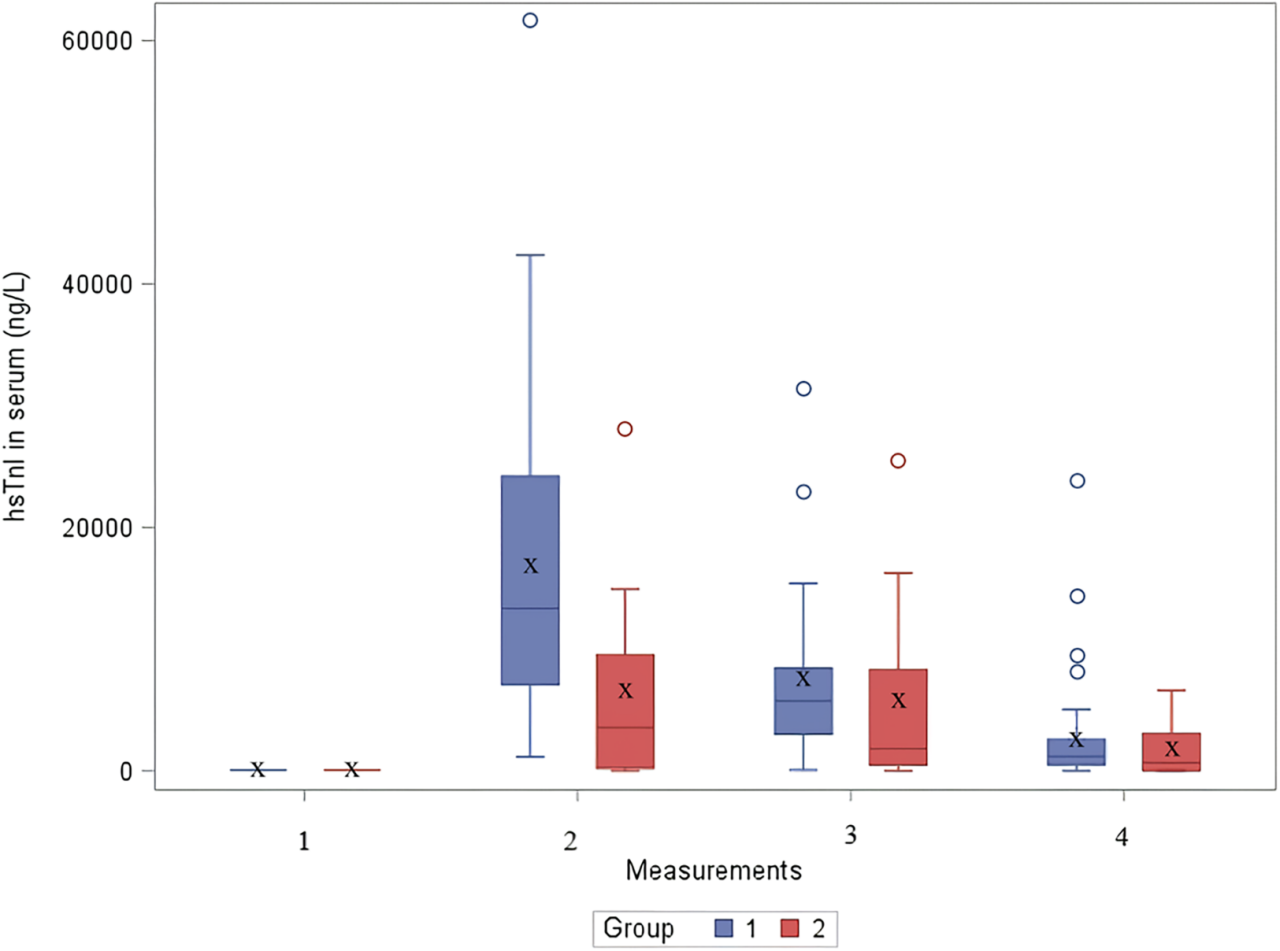
The distribution of cTnI concentration in serum by group over time. hsTnI, high-sensitive troponin I; Group 1, patients after VSD surgery; Group 2, patients after formation of BCPC anastomosis.

In the second group, the median value of cTnI before surgery in serum was 10.6 [IQR: 6.4–18] ng/L (Table 3). Immediately after the surgery (measurement 2) the the median value of cTnI was 6,905.7 [IQR: 3,083–11,780] ng/L. On the first postoperative day the median value was 2,267.4 [IQR: 1,226–8,298] ng/L, and on the third postoperative day the median value was 868 [IQR: 649–4,167] ng/L. The distribution of cTnI concentration in serum by group over time is shown in Figure 1.

Statistically significant difference using one-sided Wilcoxon Mann–Whitney test was found in the second measurement (immediately after cardiac surgery), where higher values of hsTnI were observed in a group of children after VSD surgery (*p* < 0.05) (Table 3). In other measurements, no significant difference was found.

**Figure 2:**
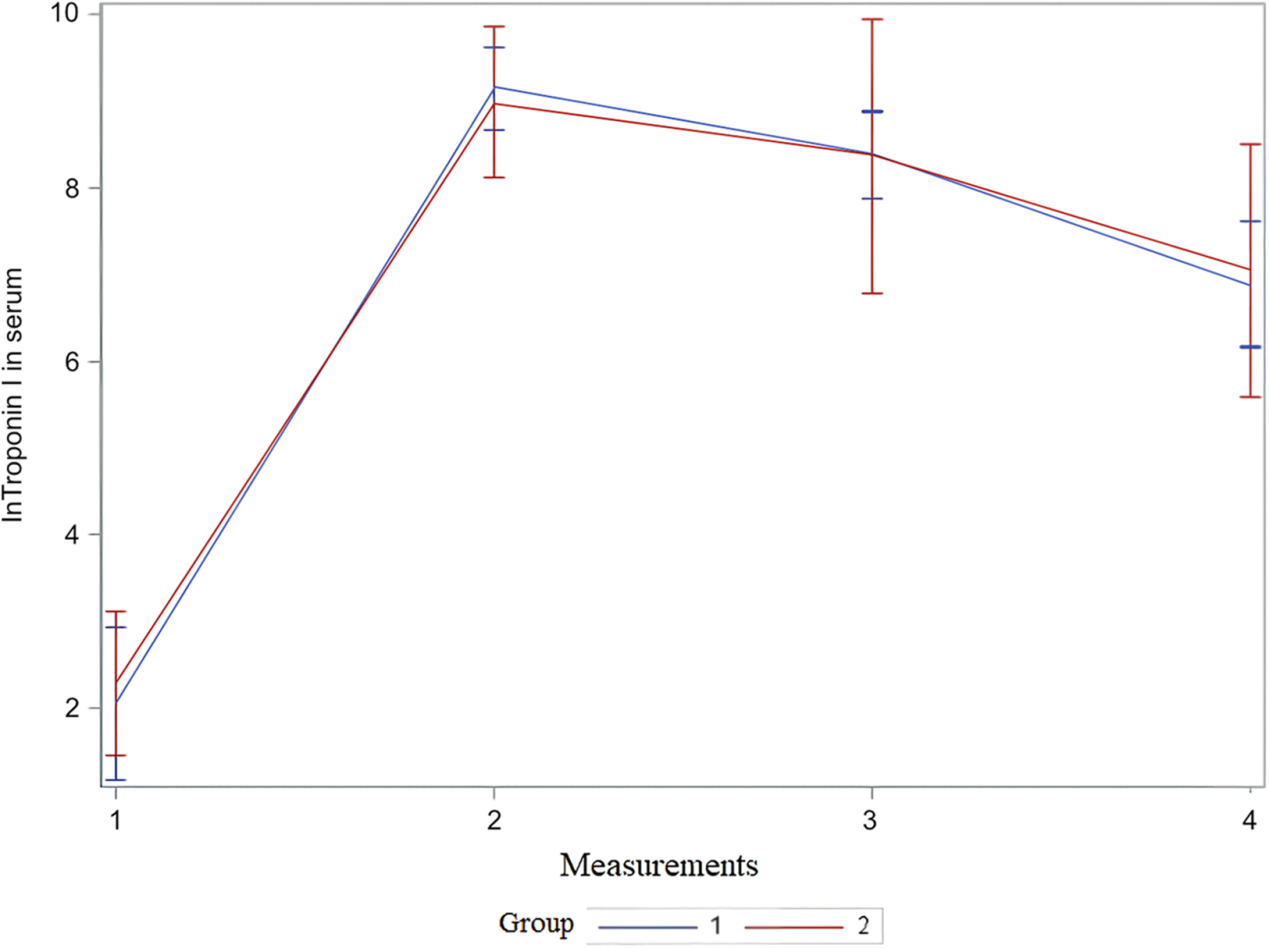
Mean and 95% CI of serum lnTroponin I value during observed period. The x-axis shows the number of measurements, and the y-axis shows the logarithmic transformation of troponin I in serum and its confidence interval. CI, confidence interval; lnTroponin I, logarithmic transformation of troponin I; Group 1, patients after VSD surgery; Group 2, patients after formation of BCPC anastomosis.

For the statistical test repeated measures analysis of variance (RM ANOVA) troponin I values were logarithmically transformed. RM ANOVA showed that there was no statistically significant difference between the groups (VSD and BCPC) for lnTroponinI (Figure 2 and Table 4). Interactions (time*group in Table 4) between the analyzed values during the period (measurements) and groups (VSD and BCPC) were not statistically significantly different. This means that lnTroponinI in serum have similar trend in the observed period in the two groups.

A negative correlation was found between lnTroponinI and creatinine clearance in the first three measurements. A positive correlation was found between lnTroponinI values in the first three measurements and PICU stay, hospital stay, hours of mechanical ventilation, cardiopulmonary bypass time, and aortic cross-clamping time. The correlation was significant in the second and third measurements for CPB time and in the third measurement for AOX time (Table 5).

### 3.3 Urine cTnI

**Figure 3:**
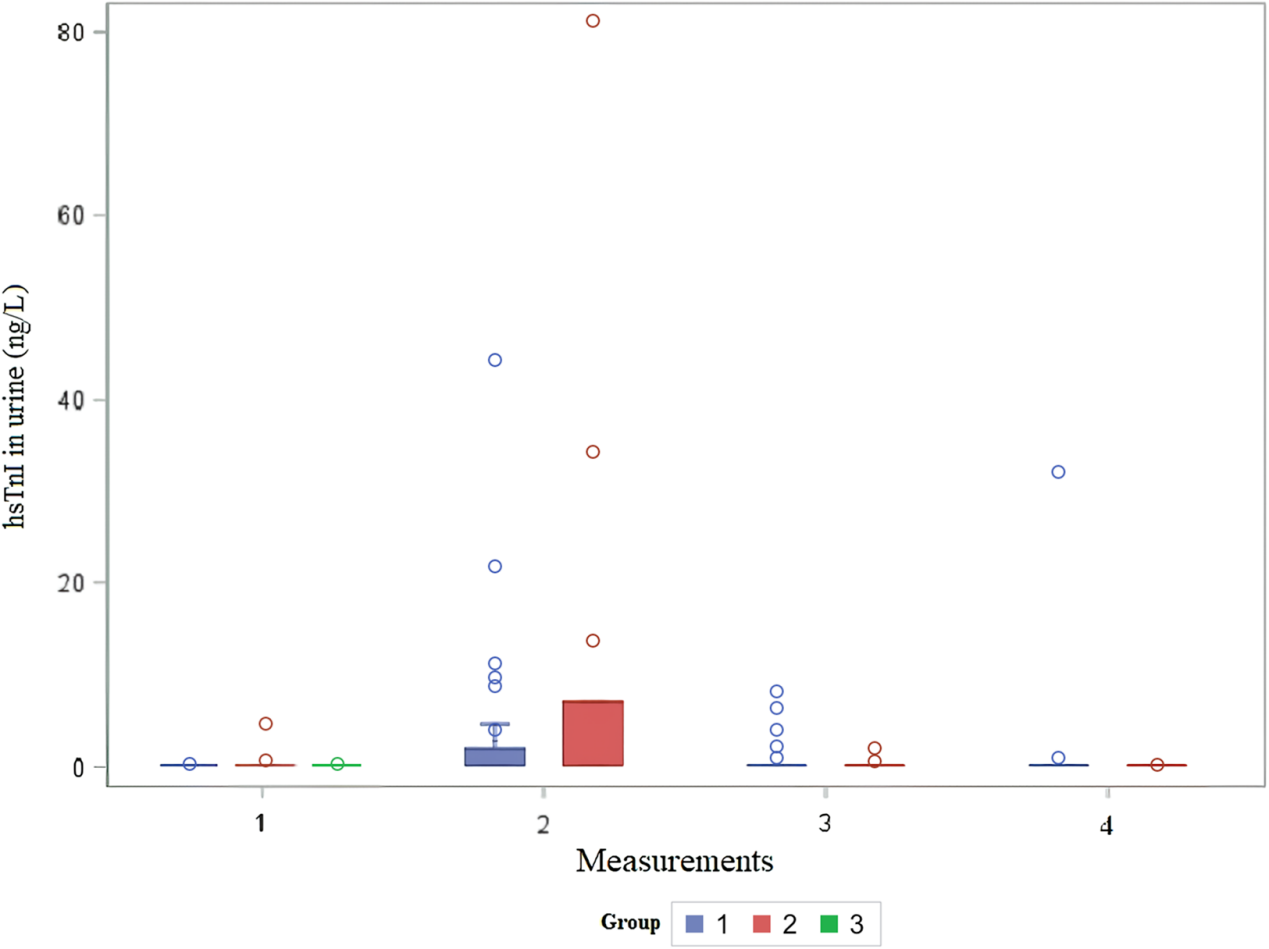
Distribution of urine hsTnI in children before and after cardiac surgery. The x-axis shows the number of measurements, and the y-axis shows the concentration of urine troponin I in ng/L per group. hsTnI, high-sensitive troponin I; Group 1, patients after VSD surgery; Group 2, patients after formation of BCPC anastomosis; Group 3, healthy control.

The distribution of cTnI in urine by group over time is shown in Figure 3. The urine cTnI was detected in 2.4% of measurements before cardiac surgery, and it was present in the patient before BCPC surgery; the concentration of cTnI in urine was 4.6 ng/L. Immediately after surgery, urine cTnI was detected in 27% of measurements (12 times); in the first group in 25% of measurements (9 times) [median 11.3 (IQR: 6.8–61.8) ng/L], and in the second group in 33% of measurements (3 times) [median 34.4 ng/L (min 7.1–max 81.2) ng/L]. On the first postoperative day, urine cTnI was measured in 13.5% of measurements (five times) in Group 1 [median 4 (IQR: 2.15–7.3) ng/L], and in 22% of measurements (two times) in Group 2 (2.1 ng/L in both patients), while on the third postoperative day, cTnI was measured in 2.8% of measurements (once) in Group 1 (32 ng/L). In healthy infants, cTnI could not be detected in any of the measurements. Patients for whom troponin levels could be assessed in urine did not demonstrate the highest troponin concentrations in their blood. Although creatinine concentrations in urine were measured, we could not express it as a ratio to the urinary creatinine concentration.

No further statistics could be provided.

## 4 Discussion

### 4.1 Troponin detection challenges in children

The primary findings of the study included anticipated higher values of cTnI in children immediately after cardiac surgery and the inability to detect cTnI in children's urine. Before the initiation of this research in 2018, only a few abstracts and papers discussed the possibility of measuring troponin T or I in urine (7, 8, 10, 18, 19). The expected increase of cTnI was also based on the study published by Pervan et al., where elevated levels of cTnI were observed in patients with hypertension compared to the healthy population (8). Pervan et al. also conducted a pilot study, providing a preliminary reference interval for cTnI in adults (9). The concept behind our study was comparable, but we had to slow down the research because of the unexpectedly challenging task of detecting cTnI in urine. The rationale for the possible detection of troponin I molecule in urine in the study mentioned above was the fact that subclinical myocardial damage can occur in patients with hypertension (20–22). Since this study was conducted on an adult population, the absence of urine troponin I detection in children in our study may be attributed to the immaturity of the kidneys. Heart failure and myocardial damage remain significant contributors to morbidity and mortality in the modern world. Despite the use of echocardiography, assessing cardiac function is often challenging, prompting numerous studies on potential biomarkers that could serve as reliable predictors of treatment response and outcome (4).

### 4.2 Troponin I in serum

The pediatric population of patients with CHD represents a particularly vulnerable surgical population. Commonly used markers for diagnosing heart damage include CK (creatine kinase), CK-MB (creatine kinase MB), myoglobin and HFABP (heart-type fatty acid binding protein), which are all found in the cytoplasm, then cTnT and cTnI and the myosin light chain that make up myofibrils (1). cTnT, cTnI and HFABP are found inside the myocytes of heart cells and can all infiltrate into the bloodstream in case of myocyte damage. In childhood, the primary biomarkers for assessing heart damage are troponin T and I. Surgical procedures for CHD are the most common cause that leads to the release and detection of heart damage markers in children. Cardiac troponins serve as high-sensitive markers for the detection of cardiac damage, revolutionizing the understanding of even minor myocardial injury (23). In the pediatric population, cardiac troponins show a strong correlation with the severity of cardiac damage following cardiac surgery, providing valuable predictive insights into recovery and mortality (2, 5, 23, 24). It has been hypothesized that troponin I may be a more specific and sensitive marker than troponin T, as troponin I remains unaffected by renal function (25). Troponin I typically peak within 24 h after CHD cardiac surgery, followed by a subsequent decrease and normalization within 5–7 days post-surgery (4). Previous research indicates that infants exhibit higher troponin values after myocardial damage in comparison with older children, influencing the decision to conduct this study specifically on children under the age of 24 months of age (26). In our study, troponin I showed a positive correlation with the duration of cardiopulmonary bypass duration and duration of aortic ischemia. However, existing literature presents conflicting results on whether troponin I values are associated with an adverse outcome after cardiac surgery (26, 27). Su et al. discovered that the persistence of troponin I values eight hours post-surgery serves as a robust and independent predictor of hypoperfusion injury and mortality (4). The clinical significance lies in the potential for additional troponin I measurements at 8- and 12-h post-surgery, offering a means to identify patients at an increased risk for postoperative mortality and morbidity when combined with other parameters (4).

### 4.3 Troponin I in urine

The challenge associated with detecting all proteins in urine, despite the advantage of its large and non-invasive collection, lies is protein identification (28). Numerous factors impede diagnosis, including high concentrations of salts and urea, a wide range of urine acidity (varying pH), and a relatively low protein concentration (29). Cardiac troponins are characterized by their presence in the bloodstream as degradation products, detectable through existing commercial tests. Although analytical cTnI immunoreactivity has been extensively studied in blood, troponin can also be detected in urine. Approximately 70% of troponin I, based on its molecular mass of 24 kilodaltons, is filtered and subsequently excreted by the kidneys (30). Currently, it remains unclear which form of the troponin molecule is present in urine and what causes immunoreactivity (30). The isoelectric points of the degradation molecules differ, for troponin C it is 4.1, for troponin I 9.9, and for troponin T 5.1 (31). The isoelectric point represents the pH value of the solution at which the net charge of the molecule is zero. Therefore, the solubility of a molecule in a certain medium can be estimated based on the isoelectric point value, which is directly dependent on the pH value of the medium itself. Although our study did not investigate the influence of urine pH on the solubility of troponin molecules, it could potentially provide insights into why troponin I cannot be measured in urine. Another possible explanation can be hidden in the size of troponin I molecule. Examination of the crystal structure of the cTnI molecule, consistently bound to troponin C in these analyses, revealed that the dimension of this complex is 50 × 30 × 30 Å (1 Ångström = 10–10 meter) (31), which may be too large to be filtered by the kidney and thus detected in the urine. A recent noteworthy study by Chen et al. (7) explored the detection of troponin I in urine as a predictor of cardiovascular events in patients with diabetes mellitus (DM). All included subjects had DM., and troponin I concentration was determined in fresh urine. Subsequently, the medical records of the subjects were prospectively monitored for three months. A total of 378 subjects were included in the study. The authors observed significantly higher urinary troponin I values in individuals who experienced subsequent cardiovascular events compared to those without such events (7). By multivariate logistic regression analysis, using different models, the value of cTnI in urine >4.10 ng/L was an independent factor for the consequent cardiovascular risk in the follow-up period of three months (7). The studied population consists of patients who are at increased risk of cardiovascular incidents due to a positive history of arterial hypertension, chronic renal failure, and heart failure with reduced or preserved ejection fraction (7). In another study involving an adult population, the authors demonstrated a significant correlation between the ratio of albumin to creatinine in urine and troponin I levels in urine among patients with chronic kidney disease, but not in those without this condition. Specifically, patients with DM and chronic kidney disease exhibited markedly higher urinary hsTnI values in cases of both micro- and macro-albuminuria (7). However, our study did not explore micro- and macroalbuminuria, and the urine troponin I detection level was exceptionally low. Consequently, we were unable to replicate similar findings to those reported in the study mentioned above, and the underlying reason for this discrepancy remains elusive.

## 5 Limitations of the study

Our study faces several limitations. Firstly, the sample size is relatively small. Secondly, the global influence of the COVID-19 pandemic may impact pre-analytical and analytical factors influencing detection. Thirdly, ethical considerations have resulted in the unavailability of blood samples from the healthy control group. Additionally, Group 2 comprises fewer subjects. Additionally, group 2 has a smaller number of subjects included.

## 6 Conclusion

Despite limitations, the inability to detect troponin I in urine is noteworthy. Future studies investigating urine troponin I should incorporate an examination of urine pH. Given that troponin I's isoelectric point falls within the alkaline range, the acidic nature of urine (low pH) may partially neutralize the negative charge of the troponin I molecule. This structural change might obscure it from immunoenzymatic tests, leading to its undetectability.

## Data Availability

The raw data supporting the conclusions of this article will be made available by the authors, without undue reservation.
